# Particulate matter exposure shapes DNA methylation through the lifespan

**DOI:** 10.1186/s13148-019-0726-x

**Published:** 2019-08-30

**Authors:** L. Ferrari, M. Carugno, V. Bollati

**Affiliations:** EPIGET—Epidemiology, Epigenetics and Toxicology Lab, Department of Clinical Sciences and Community Health, Università degli Studi di Milano, via San Barnaba 8, 20122 Milan, Italy

**Keywords:** DNA methylation, Environmental exposures, Particulate matter

## Abstract

Exposure to airborne particulate matter (PM) has been associated with detrimental health effects. DNA methylation represents the most well-studied epigenetic factor among the possible mechanisms underlying this association. Interestingly, changes of DNA methylation in response to environmental stimuli are being considered for their role in the pathogenic mechanism, but also as mediators of the body adaptation to air pollutants.

Several studies have evaluated both global and gene-specific methylation in relation to PM exposure in different clinical conditions and life stages. The purpose of the present literature review is to evaluate the most relevant and recent studies in the field in order to analyze the available evidences on long- and short-term PM exposure and DNA methylation changes, with a particular focus on the different life stages when the alteration occurs. PM exposure modulates DNA methylation affecting several biological mechanisms with marked effects on health, especially during susceptible life stages such as pregnancy, childhood, and the older age.

Although many cross-sectional investigations have been conducted so far, only a limited number of prospective studies have explored the potential role of DNA methylation. Future studies are needed in order to evaluate whether these changes might be reverted.

## Introduction

Air pollution is a composite mixture of toxicants, deriving from both natural (e.g., erosion of the earth crust and wildfires) and anthropogenic (e.g., transportation, biomass burning, and home and industrial heating) sources. The most recent Global Burden of Disease Study reported that air pollution is responsible for 19% of the overall cardiovascular mortality, including 23% of all deaths from ischemic heart disease and 21% of those from stroke [1]. Although this mixture is extremely variable across locations and seasons, particulate matter (PM) is thought to be one of its most harmful components. According to the 2018 World Health Organization (WHO) report on air pollution, more than 90% of the world population is exposed to levels of PM with diameter less than or equal to 10 or 2.5 μm (PM_10_ or PM_2.5_, respectively) exceeding the WHO air quality guidelines [2].

**Fig. 1:**
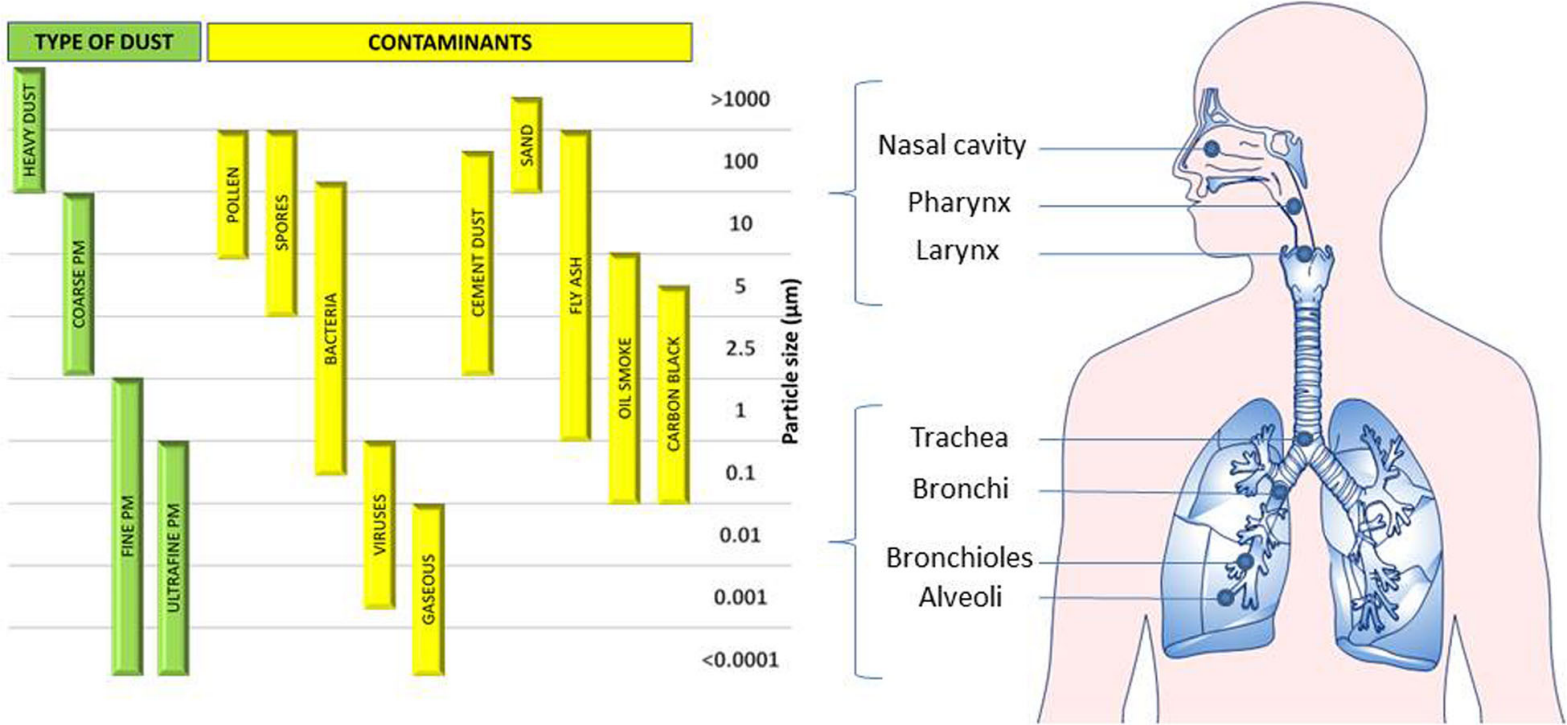
Regional deposition of inhaled particles in the respiratory tract is size-dependent. Heavy dust, coarse, fine, and ultrafine PM are constituted by different contaminants. PM enters the body through the respiratory tree, and particle dimensions influence the level of penetration in the lungs: PM with an aerodynamic diameter below 10 μm penetrates into the bronchi; PM below 2.5 μm reaches the alveoli

PM itself is not a single toxicant but rather a combination of carbon, crustal elements, heavy metals, polycyclic aromatic hydrocarbons (PAHs), and inorganic ions [3]. The size of the particles influences the level of particle penetration in the respiratory tree: PM with diameter between 2.5 and 10 μm (usually called “coarse” PM or PM_2.5–10_) can penetrate into the bronchi, while PM_2.5_ can reach the alveoli (Fig. 1). Whereas PM_10_ and PM_2.5_ are known to produce a local inflammation in the lungs, there is no conclusive evidence that complete particles enter and deposit in blood vessels. However, it seems plausible that smaller components of PM can enter the bloodstream as recent evidences showed in healthy volunteers a translocation of 10-nm inhaled gold nanoparticles into the systemic circulation and accumulation at sites of vascular inflammation [4]. In addition, it has been recently shown that black carbon particles can be detected in different kinds of peripheral compartments (e.g., urines) [5]. While exposure to PM has been consistently associated to several negative health effects, impacting mainly on the respiratory and cardiovascular system, the biological mechanisms underlying this association have been only partially elucidated. In this context, epigenetic mechanisms are thought to have a central role, not only as relevant elements of the pathogenic mechanism, but also as mediators of the body adaptation to environmental stimuli, such as air pollutants.

Epigenetic mechanisms are, in fact, flexible genomic parameters that can alter genome expression under exogenous influence but also guarantee the stable propagation of gene activity states through subsequent cell generations [6]. Alterations in epigenetic marks have also been associated with a variety of human diseases, including cancer, and cardiovascular, respiratory, and neurodegenerative disorders [7]. The most investigated epigenetic mechanism is DNA methylation, which implies the adding of a methyl group to the 5′ position of cytosine residues located in a CG dinucleotide. Generally speaking, DNA methylation in gene promoters acts as a repressor of gene expression [8], whereas an overall decrease in DNA methylation (mainly due to hypomethylation of repetitive elements and non-coding regions) is frequently observed in cancer cells and can affect genomic stability [9]. In addition, DNA methylation occurring in gene bodies is thought to be related to alternative transcript limitation and splicing control [10]. Finally, global methylation (often estimated by measuring repetitive element methylation, i.e., Alu and LINE-1) represents the overall methylation state of the genome without specifying in which genomic locations the methylation occurs [11].

Other reviews critically revised the literature in the field, but this is the first that tries to integrate the current knowledge throughout life stages [12, 13]. Indeed, the purpose of this literature review is to provide a critical analysis of the available evidence on PM exposure and associated DNA methylation changes, with a particular attention to the various life stages when the alteration occurs (Table 1). We searched PubMed (last update July 2019) to find studies on the association between particulate matter exposure and DNA methylation. We combined the MeSH term for particulate matter (i.e., MeSH Unique ID: D052638) with the MeSH terms for DNA methylation (MeSH Unique ID: D019175). The search was complemented by cross-referencing the identified studies and review articles. Although the comprehensive review of the evidences describing the epigenetic alteration occurring in disease is outside the direct focus of this paper, we will also mention the main diseases in which alteration in DNA methylation have been hypothesized as an intermediate step between PM exposure and disease development.

**Table 1:** Particulate matter effects on DNA methylation, in different life stages

Life stage	↑/↓*	Genes	Type	Tissue	Ref.	Number
Preconceptional	↑	Global	Mouse	Sperm	Yauk et al. (2008)	[14]
Pregnancy, first trimester	↑	LINE-1	Human	Blood spots	Breton et al. (2016)	[15]
Pregnancy, first trimester	↓	Global	Human	Placenta	Janssen et al. (2013)	[16]
Pregnancy, first trimester	↓	LINE-1	Human	Placenta	Cai et al. (2017)	[17]
Pregnancy	↓	LINE-1	Human	Placenta	Kingsley et al. (2016)	[18]
Pregnancy	↑↓	7 CpG sites (i.e., three located near PTPRN2, TMEM125, and VPS4A genes, the other 4 sites mapped to non-genic regions)	Human	Placenta	Kingsley et al. (2016)	[18]
Pregnancy, first and second trimester	↑	HSD11B2	Human	Placenta	Oakley and Cidlowski (2013)	[19]
Pregnancy, second trimester	↑	SOD2	Human	Cord blood and maternal blood	Zhou et al. (2019)	[20]
Pregnancy	↑↓	APEX1, OGG1, ERCC4, TP53 DAPK1	Human	Placenta	Neven et al. (2018)	[21]
Pregnancy, third trimester	↑	NPAS2, CRY1, PER2, PER3	Human	Placenta	Nawrot et al. (2018)	[22]
Pregnancy, first trimester	↑	D-loop, MT-RNR1	Human	Placenta	Janssen et al. (2015)	[23]
Childhood, asthma	↓	Immune genes (e.g., IL13 and RUNX3)	Human	Blood	Yang et al. (2015)	[24]
Childhood, asthma	↑	FOXP3	Human	Blood	Prunicki et al. (2018)	[25]
Childhood	↓	IL-4, IFN-γ	Human	Blood	Jung et al. (2017)	[26]
Childhood	↓	TET1	Human	Nasal airway cells	Somineni et al. (2016)	[27]
Childhood	↑	FAM13A, NOTCH4	Human	Blood	Gruzieva et al. (2019)	[28]
Adult age, healthy	↑↓	MATN4, ARPP21, CFTR	Human	Blood	Gondalia et al. (2019)	[29]
Adult age, obese	↓	CD14, TLR4	Human	Blood	Cantone et al. (2017)	[30]
Adult age, occupational exposure	↓	NOS3, EDN1	Human	Blood	Tarantini et al. (2013)	[31]
Adult age, CVD	↑↓	cg20455854, cg07855639, cg07598385, cg17360854, cg23599683	Human	Blood	Chi et al. (2016)	[32]
Adult age, CVD	↓	Global	Human	Blood	Plusquin et al. (2017)	[33]
Adult age, CVD	↓	Alu, TLR4	Human, crossover	Blood	Bellavia et al. (2013)	[34]
Adult age, CVD	↑	IFN-γ	Human, crossover	Blood	Tobaldini et al. (2018)	[35]
Adult age, CVD	↑↓	Loci related to insulin resistance, glucose and lipid metabolism, inflammation, oxidative stress, platelet activation, and cell survival and apoptosis	Human, crossover	Blood	Li et al. (2018)	[36]
Adult age, CVD	↑↓	Loci related to apoptosis, cell death and metabolic pathways, or associated with ion binding and shuttling	In vitro	Human cardiomyocytes AC16	Yang et al. (2018)	[37]
Adult age, respiratory disease	↑↓	2827 CpG sites (genes involved in inflammation and oxidative stress response), repetitive elements, and microRNA	Human, crossover	Blood	Jiang et al. (2014)	[38]
Adult age, respiratory disease	↑↓	12 differentially methylated probes and 27 differentially methylated regions	Human	Blood	Lee et al. (2019)	[39]
Adult age, cancer	↑	P16^INK4A^	In vitro	Ex vivo lymphocytes	Fougere et al. (2018)	[40]
Adult age, cancer	↑	P16^INK4A^	In vitro	Primary human bronchial epithelial cells	Leclercq et al. (2017)	[41]
Adult age, cancer	↑↓	66 genes	In vitro	BEAS-2B cells	Hesselbach et al. (2017)	[42]
Adult age, cancer	↑↓	P16^INK4A^, APC, LINE-1, NOS2	Mouse	Blood	Ding et al. (2016)	[43]
Adult age, cancer, DOHaD	↑↓	*SYK*, *CCND2*	Human	Blood	Callahan et al. (2018)	[44]
Adult age	↑	MT-TF, MT-RNR1	Human	Blood	Byun et al. (2013)	[45]
Adult age	↓	D-loop	Human	Blood	Byun et al. (2016)	[46]
Elderly	↑↓	Genes involved in tumor development, gene regulation, inflammatory stimuli, pulmonary disorders, and glucose metabolism	Human	Blood	Panni et al. (2016)	[47]
Elderly	↑↓	LINE-1, Alu, IL-6	Human	Blood	Bind et al. (2012)	[48]
Elderly	↓	iNOS	Human	Blood	Madrigano et al. (2012)	[49]

*Increase (↑) or decrease (↓) in DNA methylation

## Preconception

Airborne pollution is thought to be able to alter fertility by impacting gamete maturation [50].

Some studies have evaluated different qualitative parameters (e.g., morphology, motility, number) on the sperm of human males. Only one study, conducted in a highly polluted district in the Czech Republic on a cohort of 2585 parental pairs, documented a significant positive association between exposure to high air pollution and percentage of sperm with DNA fragmentation (according to sperm chromatin structure assay) [51]. No information has been reported so far about air pollution and related alterations in the DNA methylation of gametes. However, environmental factors might exert heritable effects through this type of alterations, since epigenetic modifications can be transmitted across the germ line, where DNA methylation is strictly regulated [52, 53]. Exposure to varying concentrations of air pollutants might thus result in sperm DNA damage and thereby increase the rates of male-mediated infertility, miscarriage, and other adverse reproductive outcomes.

Only studies conducted in animal models evaluated the impact of air pollution on DNA methylation of male gametes [54]. Different studies reported that air pollutants affect the quality of sperm: a significant decrease in the daily production of spermatozoa and a parallel increase in the proportion of abnormal sperm shapes have been observed in mice and rats exposed to air pollutants, especially from diesel exhaust [54]. Yauk and colleagues reported a statistically significant increase in sperm DNA breakage and global hypermethylation in mice exposed to airborne pollution from air near two integrated steel mills and a major highway in Hamilton, Ontario (Canada) [14]. In this study, global methylation was evaluated in the sperm of mice exposed to whole air or high-efficiency particulate air (HEPA)-filtered air. Mice continuously exposed to particulate air pollution for 10 weeks showed global hypermethylation, which persisted up to 6 additional weeks after cessation of exposure.

Very few studies have been investigating the impact of air pollution on female reproductive parameters in spontaneous fertilization, and no studies analyzed DNA methylation [54]. This can be explained by the greater difficulties involved in investigating female rather than male gametes. Elucidating the effects of exposure to airborne pollutants on female gametes would thus be extremely important, not only in terms of DNA methylation, but also as regards their functionality and integrity.

## Pregnancy

Adverse health outcomes which have been associated to air pollution exposure during pregnancy include events occurring during pregnancy and at birth (e.g., low birth weight, fetal growth restriction, preterm birth), or manifest later in life (e.g., neurodevelopmental disorders, reduced infant lung function) [55–61]. Air pollution exposure during pregnancy has been also associated to increased risk of pregnancy-induced hypertensive disorders, thus representing a threat for the mother as well [62].

The placenta plays a crucial role in the regulation of fetal growth and development; in addition, several studies have been clarifying that a proper epigenetic regulation of genes is important in placental growth and functioning and that toxic substances may possibly xinterfere with placental function through epigenetic alterations [63–66]. Particles smaller than 240 nm in diameter can cross the placenta to the fetal side and affect the placental function also by modifying its epigenome [67, 68]. Several investigations (as discussed below) have so far documented the association between air pollution exposure during pregnancy and alterations of placental and/or cord blood DNA methylation, with a concordantly reported hypomethylation of repetitive elements. Indeed, Breton and colleagues reported that prenatal exposure to PM_10_ (32 μg/m^3^) during the first trimester was associated with lower placental LINE-1 methylation measured in DNA extracted from 459 newborn blood spots (2-SD increase *β* = – 0.66; 95% CI − 1.22, − 0.09) [15]. In the ENVIRONAGE birth cohort, placental global DNA methylation [measured by quantifying 5′-methyl-deoxycytidine (5-mdC) and deoxycytidine (dC) using ultra-pressure liquid chromatography (UPLC), in combination with tandem mass spectrometry (MS-MS)] was inversely associated with PM_2.5_ exposure [averaged 17.4 (15.4–19.3) μg/m^3^] experienced during the whole pregnancy (− 2.19%, 95% CI − 3.65, − 0.73%, *p* = 0.004). If considering a multi-lag model, with exposures of the three trimesters fitted as independent variables at the same time, only exposure to PM_2.5_ during the first trimester was significantly associated with lower global DNA methylation (− 2.13% per 5 μg/m^3^ increase; 95% CI − 3.71, − 0.54%, *p* = 0.009). Focusing on the first trimester, the days when implantation might occur (6–21 days) appeared as the most sensitive time window (− 1.08% per 5 μg/m^3^ increase; 95% CI − 1.80, − 0.36%, *p* = 0.004) [16]. In the same cohort, elevated prenatal exposure to PM_2.5_ and black carbon was also associated with an increased placental Alu mutation rate. In addition, in a case-control study including 220 subjects, placental LINE-1 DNA methylation was inversely associated with the first trimester PM_10_ exposure (− 1.78%; 95% CI − 1.78 − 3.35, − 0.22%) [17].

In an independent cohort, Kingsley and colleagues reported that living close to a major roadway was associated with a lower birth weight and a lower mean placental LINE-1 methylation levels in fully adjusted models (95% CI − 1.57, − 0.07; *p* = 0.03) and identified seven CpG sites (i.e., three located near *PTPRN2*, *TMEM125*, and *VPS4A* genes, the other four sites mapped to non-genic regions) significantly associated with this residential proximity [18].

Methylation levels associated with some genes can vary throughout pregnancy, as DNA methylation could have specific patterns depending on gestational age (e.g., highly methylated in the first trimester and then hypomethylated): the effect of pollutant exposure might thus be dependent on the exposure window considered. Cai and colleagues evaluated the placental methylation levels of *HSD11B2*, encoding the 11β-hydroxysteroid dehydrogenase 2, which is involved in glucocorticoid metabolism and has a critical role in fetal growth [19]. *HSD11B2* methylation levels were positively associated with both the first and second trimester PM_10_ exposure (first trimester 1.03%, 95% CI 0.07, 1.98%; third trimester 22.33%, 95% CI 0.69, 3.76%). The association was more evident in newborns who experienced intrauterine growth restriction than in normal weight newborns, thus suggesting that the alteration of *HSD11B2* methylation might contribute to PM-induced reproductive and developmental toxicity [17].

Zhou and colleagues investigated the effects of prenatal PM exposure on superoxide dismutase 2 (*SOD2)* methylation, as it is responsible for detoxifying superoxide radicals, preventing oxidative injury that leads to many diseases, such as tumors, obesity, and cardiovascular and neurological diseases. To this aim, they measured promoter methylation levels in the peripheral blood of 568 pregnant women and in the umbilical cord blood from their newborn from Houzhai Town, China. They observed that *SOD2* methylation in both maternal peripheral blood (*β* = 2.19, *p* = 0.029) and umbilical cord blood (*β* = 2.69, *p* < 0.001) were positively associated with PM_10_ exposure concentrations during the entire pregnancy, and in particular during the second trimester (*p* < 0.001 for both maternal and umbilical cord blood). Moreover, *SOD2* promoter methylation in the umbilical cord blood and PM_10_ exposure during the entire pregnancy (13.5% [95% CI 4.2, 35.7]) and the second trimester (9.4% [95% CI 2.8, 27.1]) were partly mediated by maternal *SOD2* promoter methylation [20]. Since exposure to particulate air pollution has been also linked with carcinogenic risk, Neven and colleagues evaluated the methylation levels of key placental DNA repair genes and reported that air pollutants may induce changes to fetal and neonatal DNA repair capacity, as well as affect tumor suppressor genes. The study, performed within the ENVIRONAGE cohort, showed a positive association between PM_2.5_ levels (increments 3.84 μg/m^3^; *r* = 0.26, *p* < 0·0001) and promoter methylation of *APEX1* (7.34%, 95% CI 0.52 to 14.16, *p* = 0.009), *OGG1* (13.06, 3.88 to 22.24, *p* = 0.005), *ERCC4* (16.31%, 5.43 to 27.18, *p* = 0.01), and *TP53* (10.60%, 4.46 to 16.74, *p* = 0.01), whereas promoter methylation of *DAPK1* (− 12.92%, − 22.35 to − 3.49, *p* = 0.007) was inversely associated with PM_2.5_ levels. In addition, black carbon exposure was found to be associated with hypermethylation of *APEX1* (9.16%, 4.06 to 14.25, *p* = 0.01) and *ERCC4* (27.56%, 17.58 to 37.55, *p* < 0.0001) promoters [21].

Airborne pollution has also been associated with changes in the methylation status of the circadian pathway genes. The circadian pathway is an important molecular target for healthy development, since a 24-h period central biological clock maintains in mammals the daily rhythm in accordance with the external environment. In order to evaluate the role of PM_2.5_ exposure on the methylation of the circadian genes, Nawrot et al. quantified, in 407 newborns, the placental methylation of CpG sites within the promoter regions of *CLOCK*, *BMAL1*, *NPAS2*, *CRY1-2*, and *PER1-3* genes.In a multi-gene model, placental circadian pathway methylation was positively and significantly associated (*p* < 0.0001) with the third trimester PM_2.5_ exposure. The single-gene models showed relative methylation differences in placental *NPAS2* (+ 0.16; *p* = 0.001), *CRY1* (+ 0.59; *p* = 0.0023), *PER2* (+ 0.36; *p* = 0.0005), and *PER3* (+ 0.42; *p* = 0.0008) for exposure during the third trimester as well [22].

The effects of airborne pollution exposure which were associated also with preterm birth have been previously reviewed by Lin and colleagues [65]. However, results from studies vary greatly and are not conclusive due to the small number of studies and their limits. To our knowledge, no studies have evaluated DNA methylation levels in association with PM exposure and preterm birth. Nonetheless, given the biological plausibility of the association between air pollution and preterm birth, and that DNA methylation is a well-established biomarker for PM exposure, further studies should be encouraged in this field as they may enable the identification of epigenetic markers, allowing for earlier detection of women at risk for delivering preterm.

## Childhood

Although all life stages can be affected by adverse health effects of air pollution exposure, children’s vulnerability is unique. Starting from the observation that the lungs keep developing during childhood, the link between air pollution and DNA methylation in children has been mainly correlated with allergic respiratory diseases. Moreover, children are predominantly oral breathers, meaning that the primary nasal filter is by-passed and polluted particles can enter the lower airways. Most of the studies (as discussed below) conducted on pediatric patients were thus aimed at evaluating both global and gene-specific DNA methylation as possible mediators of the association between airborne pollution and asthma exacerbation.

Yang and colleagues compared DNA methylation patterns and gene expression in inner-city children with persistent atopic asthma (*n* = 97) and in healthy control subjects (*n* = 97) by analyzing DNA from peripheral blood mononuclear cells (PBMCs). They identified 81 differentially methylated regions. Among asthmatic patients, 11 differentially methylated regions were associated with higher serum IgE concentrations, and 16 were associated with forced expiratory volume in 1 s (FEV1). In addition, in asthmatic subjects, several immune genes were hypomethylated, including *IL13* and *RUNX3*, which are genes specifically relevant to T lymphocytes [24].

Hew and colleagues evaluated the association between exposure to air pollutants and asthma in a cohort of 256 subjects from Fresno, CA, USA. Their very first investigation was indeed not focused on PM, as ambient polycyclic aromatic hydrocarbon (PAH) concentrations (ng/m^3^) were measured using a spatiotemporal regression model over multiple time periods. Higher average PAH exposure was significantly associated with increased methylation in *FOXP3* locus of DNA from PBMCs. Another study documented that these epigenetic modifications were significantly linked to differential protein expression of *FOXP3*, encoding a transcriptional regulator that is crucial for the function of regulatory T cells [69]. Methylation was also associated with functional cellular changes, including regulatory T cell dysfunction and increased plasmatic IgE levels. Finally, increasing levels of PAH were associated with decreased protein expression of IL-10 and increased expression of IFN-γ in a population of 256 subjects (including 171 non-asthmatic and 85 asthmatic subjects); the association strengthened when moving from 24-h to 1-year PAH exposure, thus suggesting a long-term effect [70]. In a subsequent study from the same research group, *FOXP3* methylation was positively associated with NO_2_, CO, and PM_2.5_ exposures at 90 days prior to the blood draw. They also observed a negative association between average *FOXP3* methylation and activated regulatory T cell levels and a positive association between average IL-10 methylation and IL-10 cytokine expression [25].

Another investigation conducted in New York City, NY, USA, showed how exposure to vanadium (considered as a trace metal component of PM), but not to PM_2.5_, was associated with lower DNA methylation of IL-4 (− 0.80, 95% Cl 0.65–0.98, *p* < 0.05) and IFN-γ (− 0.81, Cl 0.67–0.98); 6-day integrated levels of air pollutants were measured from homes of 163 children (ages 9–14), and repeated 6 months later [26]. The same authors determined also the effects of black carbon (BC) exposure on DNA methylation of pro-inflammatory genes linked to airway inflammation in asthmatic subjects. They observed that higher levels of BC were associated with lower methylation of IL4 promoter CpG-^48^ 5 days later [71].

5-Hydroxymethylcytosine (5-hmC) and *TET1* expression are known to be associated with in-house dust mite-induced asthma in the lungs of mouse models [72]. TET proteins catalyze methylation through modification of 5-methylcytosine to 5-hmC. Both *TET* methylation and 5-hmC levels were therefore evaluated in association with asthma and traffic-related air pollution in the DNA derived from nasal airway epithelial cells of 12 African American children with asthma, of their non-asthmatic siblings, and of children from an independent population (*n* = 186). Loss of methylation at a single CpG site in the *TET1* promoter and increased global 5-hmC levels were significantly associated with asthma. On the contrary, traffic-related air pollution exposure significantly increased methylation at the same site, suggesting a possible role of *TET1* methylation, still to be functionally elucidated, as a modifier of the response to traffic-related air pollution in asthmatic patients [27].

Very recently, an epigenome-wide meta-analysis [28] was conducted on nine European and American studies participating in the Pregnancy and Childhood Epigenetics consortium (PACE) [73] to evaluate the effects of prenatal PM exposure on lung-related outcomes during childhood. Six CpGs resulted to be significantly associated [false discovery rate (FDR) < 0.05] with prenatal PM_10_ and 14 CpGs with PM_2.5_ exposure. In particular, two of the PM_10_-related CpGs mapped to *FAM13A* (cg00905156) and *NOTCH4* (cg06849931) genes associated with lung function and asthma, and both CpGs were significant (*p* < 0.05) in 7- to 9-year-olds, although only the direction of the association of the CpG in *FAM13A* was consistent.

## Adult age

As described in the present section, the large majority of studies conducted so far has been focused on the adult age, most likely because adult populations are usually easier to recruit and investigate than newborns or children.

A very recent study conducted by Gondalia and colleagues identified PM-sensitive CpG sites mapped to neurological, pulmonary, endocrine, and cardiovascular disease-related genes in a very large American population of 8397 healthy subjects, enrolled within the Women’s Health Initiative (WHI) and the Atherosclerosis Risk in Communities study (ARIC) cohorts, characterized by sociodemographically and environmentally diverse features [29]. The authors evaluated genome-wide methylation levels in peripheral blood leukocytes and reported associations between PM and methylation levels of three CpG islands. PM_10_ was positively associated with cg19004594, mapping within *MATN4* gene (*p* = 3.33 × 10^−8^), which encodes Martilin 4 protein, involved in cardiac remodeling and in ematopoietic cell proliferation. PM_10_ and PM_2.5–10_ were positively associated with cg24102420, on *ARPP21* (*p* = 5.84 × 10^−8^), encoding the cAMP Regulated Phosphoprotein 21 that is involved in the regulation of calmodulin signaling. PM_2.5–10_ exposure was inversely associated with cg12124767 on *CFTR* gene (*p* = 9.86 × 10^−8^), which encodes the cystic fibrosis transmembrane conductance regulator, mainly involved in the pathogenesis of cystic fibrosis, but its function is reduced also due to environmental exposure, such as tobacco smoke [74].

PM exposure has been linked to a variety of conditions and diseases, among which cardiovascular, respiratory, and (more recently) neuropsychiatric diseases are the most represented. A common underlying mechanism is the increase in inflammation processes triggered by PM, especially by variations in its levels occurring in a short time lag.

### Cardiovascular diseases

The observation that PM exposure was associated to cardiac and cardiovascular diseases is well consolidated. Short-term exposure to PM has been linked to an aberrant methylation of several specific genes. For instance, in a study of 186 obese subjects, PM exposure resulted in an inverse association with methylation of inflammatory genes (i.e., CD14 and TLR4) while no association was observed for methylation of TNF-α [30]. Metal-rich PM exposure occurring in an occupational setting (a steel plant) was associated with NOS3 (nitric-oxide-synthase-3) and EDN1 (endothelin-1) methylation. These alterations were also linked to endogenous thrombin potential (ETP) (PM_10_ *β* = 20.0, 95% CI 3.0, 37.0; PM_1_ *β* = 80.8, 95% CI 14.9, 146.7), a global functional assay that describes overall coagulability, thus supporting the hypothesis that this work setting represents a well-characterized prothrombotic exposure [31].

The effects of 1-year ambient air pollution exposure on DNA methylation were evaluated in PBMCs of adults of the Multi-Ethnic Study of Atherosclerosis (MESA) cohort. Long-term ambient air pollution exposure was associated with DNA methylation occurring in five specific sites (cg20455854, cg07855639, cg07598385, cg17360854, and cg23599683), but not with global DNA methylation: these modifications may provide insights in clarifying the role of environmental factors in the pathogenesis of complex diseases like atherosclerosis [32]. More recently, a study conducted by Plusquin et al. investigated the effects of long-term exposure to air pollutants on average DNA methylation at functional regions and on differentially methylated individual CpG sites of samples from two large independent prospective cohort studies (the EPIC cohort and the EnviroGenoMarkers project): its main result supports the observation of a global hypomethylation associated with air pollution [33].

A similar approach was also applied on three independent studies—KORA F3 (2004–2005) and F4 (2006–2008) in Germany and the Normative Aging Study (1999–2007) in the USA—where genome-wide DNA methylation proportions were measured by means of the Illumina 450 k BeadChip. The effect of PM concentration was first assessed in each single study, with subsequent pooling of the study-specific results via meta-analysis. Twelve CpGs were associated with PM concentration (daily lags) at a genome-wide Bonferroni significance level (*p* ≤ 7.5E−8); 9 out of these 12 sites expressed increased methylation (see below for further details) [47].

All the abovementioned studies are associative in nature, and a few interesting investigations tried to overcome this limitation by creating an experimental setting (following a randomized controlled crossover study design) where healthy volunteers were artificially exposed to air particles. In one of them, 15 healthy adults were exposed to fine or coarse concentrated ambient particles (CAPs) or to HEPA-filtered medical air (control) for 130 min. Alu repetitive element methylation was lowered by fine CAPs while coarse CAP exposure lowered Toll-like receptor (TLR) 4 methylation (*β* standardized = − 0.74, adjusted *p* = 0.03). Moreover, decreased levels of Alu and TLR4 methylation were associated with higher post-exposure diastolic and systolic blood pressure [34]. In a similar experimental setting, 12 healthy subjects were exposed, through inhalation, to a filtered air mixture or to filtered air containing particulate mixture (PM_10_, PM_2.5_, PM_1_, and PM_0.5_); blood samples were collected at baseline (T1), after air exposure (T2), and after 2 h (T3) for each subject. In the PM-exposed group, a significant increase of IFN-γ methylation, from T1 to T3, was observed. Moreover, IFN-γ methylation was associated to parasympathetic modulation [35]. Finally, a randomized, double-blind, crossover trial was conducted on 36 healthy young adults in Shanghai, China, whose dormitory rooms were alternatively equipped with real and sham air purifiers for 9 consecutive days, to mimic low and high natural exposure scenarios of PM_2.5_; genome-wide methylation was then analyzed with the Illumina Infinium Human Methylation EPIC BeadChip (850 k). Between the high and low exposure groups, methylation levels were significantly changed in 49 CpG loci: 31 of these were implicated in insulin resistance, glucose and lipid metabolism, inflammation, oxidative stress, platelet activation, and cell survival and apoptosis, thus reinforcing the hypothesized role of epigenetics in the development of cardiovascular and metabolic diseases [36].

In addition, the hypothesis that PM is able to modulate DNA methylation impacting on the cardiovascular system has been explored also through in vitro models. Human cardiomyocytes AC16 were treated with PM_2.5_, and DNA methylation changes were analyzed using Illumina HumanMethylation 450 K BeadChip. PM_2.5_ was found to induce genome-wide variation of DNA methylation, particularly in genes linked to apoptosis, cell death, and metabolic pathways, or associated with ion binding and shuttling [37].

### Respiratory diseases

Respiratory diseases, such as asthma, have been also linked to PM exposure [75]. Although many studies investigated asthmatic children (see above), only a few were focused on adults.

Diesel exhaust, one of the major contributors to fine PM in urban settings, has been associated with variations in DNA methylation levels at CpG sites across the genome in circulating blood in asthmatics. In particular, a double-blind crossover study of filtered air and diesel exhaust exposures was performed on 16 non-smoking asthmatic subjects, collecting samples pre-exposure and 6 and 30 h post-exposure. DNA methylation at 2827 CpG sites (mainly related to genes involved in inflammation and oxidative stress response), repetitive elements, and microRNA were affected by diesel exhaust exposure [38].

On the contrary, in a large genome-wide methylation study conducted on the LifeLines Cohort (1017 subjects) and replicated in two independent samples from the KORA study, no significant associations were found for PM exposure (considering all different size fractions) and DNA methylation, even if several associations were found for exposure to nitrogen dioxide (NO_2_). Nonetheless, the relatively small range of PM levels and the consequent modest exposure contrast in the LifeLines cohort may partly explain this lack of association [76].

Genome-wide methylation profiles were recently evaluated in a Korean cohort of 100 subjects including 60 individuals with chronic obstructive pulmonary disease (COPD) [39]. Twelve differentially methylated probes (DMPs) and 27 differentially methylated regions (DMRs) resulted to be associated with PM_10_ long-term exposure (i.e., prediction model estimated the annual average PM concentrations in 2010). Moreover, 45 DMPs and 57 DMRs were related to NO_2_. Of the 27 DMPs associated with NO_2_ (FDR < 0.05) in the study, 11 were reported to be related to NO_2_ exposure in the LifeLines cohort [76]. On the contrary, the twelve DMPs related to PM_10_ (FDR < 0.05) were newly identified.

### Mental disorders

In the very last years, the finding that PM exposure is also associated with mental health is becoming more consistent [77–79]. We recently reviewed the available evidences supporting the possible role of air pollution in triggering mental disorders, in particular major depression [80]. The investigation of intermediate molecular mechanisms of mental disorders, in particular epigenetics, is still in its infancy. The available studies have been reviewed by Gescher and colleagues [81]. However, studies investigating the full process linking PM exposure to DNA methylation changes to mental disorder development are still lacking and will be a pressing priority in the future.

### Cancer

Exposure to environmental pollution has been also associated with increased risk of cancer. Multiple alterations in DNA methylation, including global DNA hypomethylation and gene-specific hyper- and hypomethylation, have been linked both to PM_2.5_ and PM_10_ exposure [82] and to cancer phenotypes [83]. For instance, the tumor suppressor gene P16^INK4A^ is frequently hyper-methylated in cancers [84]. Interestingly, P16^INK4A^ promoter methylation was reported to be significantly increased in PBMCs after exposure to PM [40].

Different studies have been performed, prevalently on breast and lung cancers. White and colleagues reported that PAHs were associated also with tissue hypo- and hypermethylation at multiple promoter regions such as *CCDN2*, *BRCA1*, *DAPK*, and *HIN1*, in a population of 1508 breast cancer cases and 1556 controls [85]. Exposure to air pollution during early life was also associated with an increased risk of breast cancer development later in life [85, 86]. In order to highlight some of the elements underlying this evidence, Callahan and colleagues evaluated the association of early life exposure to traffic emissions with patterns of DNA methylation in breast tumors. The study was conducted in a population of women enrolled in the Western New York Exposures and Breast Cancer (WEB) Study (*n* = 1170) [87]. Traffic emissions at menarche were associated with increased methylation of *SYK* (OR = 2.37, 95% CI 1.05–5.33), while traffic emissions at the first birth and 10 years prior to diagnosis were associated with decreased methylation of *CCND2* (OR 10 years prior to diagnosis = 0.48, 95% CI 0.26–0.89) [44].

n vitro studies conducted on primary human bronchial epithelial cells derived from either healthy subjects or chronic obstructive pulmonary disease patients revealed that exposure to PM_2.5_ from air pollution caused global DNA hypomethylation, P16 gene promoter hyper-methylation, and changes in site-specific methylation, acetylation, and phosphorylation of histone H3 (i.e., H3K4me3, H3K9ac, H3K27ac, and H3S10ph) [41]. Moreover, methylome and transcriptome analysis of PM_2.5_-induced (100 μg/ml) BEAS-2B cells identified 66 differentially expressed genes (either hyper- or hypomethylated), involved in lung diseases (particularly lung cancer) [42]. Another study observed gene methylation in healthy mice exposed to traffic-associated air pollution, including upregulation of p16 and adenomatous polyposis coli (APC) methylation and downregulation of LINE-1 and nitric-oxide-synthase-2 (NOS2) methylation [43].

## The elderly

The aging process makes the elderly more susceptible to many health threats, including short- and long-term exposure to ambient air pollution.

The two most studied elderly cohorts are from the USA (The Veteran Affairs Normative Aging Study [NAS]) [88, 89] and Germany (The KORA cohort) [90], and the relationship between air pollution and DNA methylation has been extensively investigated.

Epigenome-wide analysis conducted on KORA and NAS populations allowed to identify 12 CpG sites associated with cumulative exposure to ambient particles up to a month. Specifically, nine CpG sites

displayed increased methylation and three decreased methylation after exposure to fine ambient particle concentrations. The identified genetic loci highlight several biological pathways such as tumor development as well as gene regulation, inflammatory stimuli, pulmonary disorders, and glucose metabolism [47].

It is known that low methylation levels of LINE-1 and high methylation levels of Alu sequences are associated with higher risk of cardiovascular events in peripheral blood leukocytes [91, 92], and this evidence was confirmed also in the NAS population [48]. The air pollution effects were also associated with markers of coagulation, inflammation, and endothelial function, further supporting an association with CVDs in the elderly. As a matter of fact, particle number and black carbon were negatively associated with 12% reduction of *F3* methylation (95% CI − 17 to − 6%), while higher sulfate and ozone concentrations were related to ICAM-1 hypomethylation [93]. A positive association was reported between traffic-related pollutants and IL-6 methylation and a negative association between ozone and TLR-2 methylation [93]. Sub-chronic exposure to traffic-related pollutants was associated with significantly reduced lung function: sub-chronic exposures to air pollutants from 3- to 28-day moving averages resulted to be significantly associated with lower forced vital capacity (FVC) and forced expiratory volume in 1 s (FEV1) (1–5% lower per IQR increase in air pollution concentrations). Moreover, the associations with 28-day moving average exposures were stronger among participants with lower methylation levels in one of five CpG sites evaluated for the *TLR2* gene (position 2) and among participants with higher methylation in *GCR*, *TLR2* (position 5), *F3* (position 1), and *IL6* (position 2), suggesting that methylation in inflammation- and immunity-related genes might contribute to the adverse effects of air pollution [48, 94].

In the NAS population, *iNOS* methylation levels were decreased after acute exposure to both black carbon and PM_2.5_. Interestingly, subjects with low optimism and high anxiety had associations that were three to four times stronger than those with high optimism or low anxiety, suggesting that poor psychological functioning might enhance the association between air pollution and DNA methylation [49].

Exposure to air pollution seems to also influence biological aging [95]. The association between accelerated biological aging and air pollution was evaluated in the KORA F4 cohort [96], in which an interquartile range (0.97 μg/m^3^) increase in PM_2.5_ was associated with a 0.33-year increase in extrinsic epigenetic age acceleration (CI = 0.01, 0.64; *p* = 0.04). Traffic exposure was associated with DNA methylation age acceleration and intrinsic epigenetic age acceleration in women, while accelerated biological aging was inversely associated with black carbon in men. This latter association was confirmed also in the NAS population. Long-term exposure to air pollution seems therefore to be associated with biological aging measures in a sex-specific manner.

## Mitochondrial DNA methylation and PM exposure

The mitochondrion is a crucial target of oxidative stress in response to exogenous stimuli. Mitochondria contain DNA molecules (mtDNA) that are independent from nuclear DNA and use distinct epigenetic machinery to regulate mtDNA methylation. Effects of PM on mtDNA damage, such as copy number variation, 8-hydroxy-2′-deoxyguanosine formation, and heteroplasmy, have been investigated both in human and animal studies [97–99]. Only few studies have evaluated mtDNA methylation levels in association with PM exposure during pregnancy and during adult age.

Janssen and colleagues evaluated mtDNA methylation in the placental tissue from 381 mother-newborn pairs that were enrolled in the ENVIRONAGE birth cohort [23]. The analysis was conducted in the *D-loop* control region and 12S rRNA (*MT-RNR1*). They reported that PM_2.5_ exposure in the first trimester of pregnancy was associated with an increase in mtDNA methylation of 1.27% (95% CI 0.23%, 2.32%) in the *MT-RNR1* region and 0.44% (95% CI 0.12%, 0.75%,) in the *D-loop* region, respectively. They reported also that *MT-RNR1* methylation mediated an inverse association between PM_2.5_ [54% (95% CI 31%, 60%)].

Byun and colleagues evaluated DNA methylation levels from buffy coats in 40 male participants (20 high, 20 low exposure) from each of three different studies on airborne pollutants. The analysis was conducted by measuring DNA methylation from buffy coats of the participants. The analysis was performed on mtDNA *D-loop* region and genes essential for ATP synthesis (*MT-TF* and *MT-RNR1*). The study on steel workers, exposed to metal-rich particulate matter (measured as PM_1_) in Italy, showed that high metal-rich PM_1_ exposure was associated with higher *MT-TF* and *MT-RNR1* methylation than low-exposed controls (difference = 1.41, *p* = 0.002). *MT-TF* and *MT-RNR1* methylation was associated with PM_1_ exposure (*β* = 1.35, *p* = 0.025); moreover, *MT-RNR1* methylation was positively correlated with mtDNA copy number (*r* = 0.36; *p* = 0.02). No association were observed in the other two studies including workers on gas-station attendants exposed to air benzene in Italy and truck drivers exposed to traffic-derived elemental carbon in China [45].

The same research group investigated mtDNA methylation also in 48 healthy men working as Boilermakers in Massachusetts, USA, and evaluated blood mtDNA methylation in the mtDNA methylation in the *D-loop* promoter was associated with PM_2.5_ levels (*β* = − 0.99%, SE = 0.41, *p* = 0.02), while *MT-TF* and *MT-RNR1* methylation was not. Moreover, D-loop promoter methylation was significantly associated with markers of heart rate variability [46].

## Combination of different environmental factors

As PM is a widespread pollutant, the possibility that additional environmental factors working in combination with PM in determining the epigenetic pattern is quite relevant.

The study of more than one exposure at the same time (the so called exposome [100]), while representing a challenge in terms of study design, is certainly closer to the “real world” exposure and needs to be encouraged.

An example of multifactorial investigation is given by the combined study of PM exposure and diet. Toll-like receptor 2 (TLR2) methylation and its dietary modulation by flavonoids and methyl nutrients have been shown to modify the effect of PM_2.5_ exposure on heart rate variability [101]. In a similar study, Barchitta et al. investigated how the combination of Mediterranean diet and PM exposure might have a combined effect on LINE-1 methylation: the authors found that higher monthly PM_10_ exposure decreases LINE-1 methylation levels while the adherence to a Mediterranean diet increases them and could thus counteract the negative effect of PM_10_ exposure [102].

## Is it possible to mitigate the detrimental effect of PM on DNA methylation?

A very interesting hypothesis has been raised by a recent work by Zhong and colleagues. Following the observation that acute exposure to PM modifies DNA methylation, they conducted a crossover trial to determine whether B vitamin supplementation might contribute to moderate such changes. Ten healthy adults blindly received a 2-h-controlled exposure experiment to sham under placebo, PM_2.5_ (250 μg/m^3^) under placebo, and PM_2.5_ (250 μg/m^3^) under B vitamin supplementation (2.5 mg/day folic acid, 50 mg/day vitamin B_6_, and 1 mg/day vitamin B_12_), respectively. Epigenome-wide methylation of peripheral CD4+ T-helper cells was profiled before and after each experiment: while PM_2.5_, as expected, induced methylation changes in genes involved in mitochondrial oxidative energy metabolism, B vitamin supplementation prevented these changes. This study is very small and has several limitations, as commented by Lucock and colleagues [103], but it might open the path to preventive interventions to minimize the adverse health effects of air pollution.

## Limitations of the present investigation and future perspectives

According to the Barker hypothesis or of the Developmental Origins of Health and Disease (DOHaD), in utero exposures to different stimuli can metabolically alter the fetus and result in chronic diseases later in life. This hypothesis was first formulated to explain the association between maternal malnutrition during pregnancy and the development of coronary heart disease in the offspring [104]. From this first evidence, many different exposures have been linked to DOHaD, and recently also a role for air pollutants has been proposed [105].

Although the link between PM exposure and DNA methylation is becoming increasingly consistent, several issues make the interpretation of study results quite challenging.

First, the majority of the revised studies shows associations and does not allow to evaluate the causal relationship between air pollution exposure and the observed changes in DNA methylation. In this context, intervention studies might also play a pivotal role, as they allow to interpret findings within the framework of causal inference. However, the few experimental studies conducted in controlled environments have been performed on a very small number of subjects and might therefore lack the power to detect an association, if present.

Second, very often the changes are not reported in a standardized way, making the comparison of different studies almost impossible. The difficult interpretation of results is due not only to a lack of standardization, but also to the nature of PM, which is a complex mixture of particles and whose composition is profoundly related to the geographical area in which the study is conducted, to the presence of urban/rural areas, and to the season in which the samples were collected. A step toward a better understanding might be made by taking into account at least the major components (e.g., elemental and organic carbon, metals, and organic component) rather than limiting the exposure characterization to the total mass. Third, when considering the changes in DNA methylation reported in the studies we reviewed in the present paper, it is noticeable how some estimates are often small. Some factors can strongly impact on the biological relevance of observed methylation changes and the interpretation of these estimates. The majority of studies has been conducted on blood, and therefore, a small change in methylation might be suggestive of a larger change in methylation occurring in target tissues. Moreover, sometimes the estimates are reported for increases of 1 unit of PM (usually 1 μg/m^3^ increase) therefore representing a very small increase in pollutant concentration.

Fourth, notwithstanding the tissue specificity of DNA methylation, epidemiological studies can be conducted mostly on minimally invasive samples (e.g., blood). The majority of the studies in the field have been conducted as bulk analysis. However, whole blood itself is a mixture of different cell types, and methylation changes might thus be explained by changes in inflammation; also, alterations occurring in an underrepresented cell type might be underestimated [106]. Another critical issue is how methylation dynamics on different genomic loci converge to determine the biological identity of each cellular sub-population. Numerous strategies have been developed to overcome confounding by cell composition. The most direct method is to fractionate leukocytes and either to study a single cell type or to perform single-cell methylome analysis [107], or alternatively, to statistically adjust for directly measured cell counts or proportions [108, 109]. Therefore, future studies should consider these approaches to empower the results obtained and to better understand the molecular mechanisms impacted by PM exposure.

Finally, DNA methylation results from modifications that occur during the entire lifespan and are affected by exposure to several factors acting on DNA with different kinetics; as they do not follow a single exposure acting on a limited temporal window, this further increases the complexity of the overall interpretative framework.

In this context, a major future goal of research investigating the effects of PM exposure on human health through modifications of DNA methylation is to understand whether the changes consistently observed in DNA methylation are predictive of future risk or rather represent a mirror of DNA plasticity in response to environmental exposures (i.e., a form of adaptation). A comprehensive and integrated approach to PM-associated changes in DNA methylation could contribute to provide the rationale for intervention campaigns aimed at reducing health risks, especially in hyper-susceptible subjects, with a massive impact on public health.

## Conclusions

**Fig. 2:**
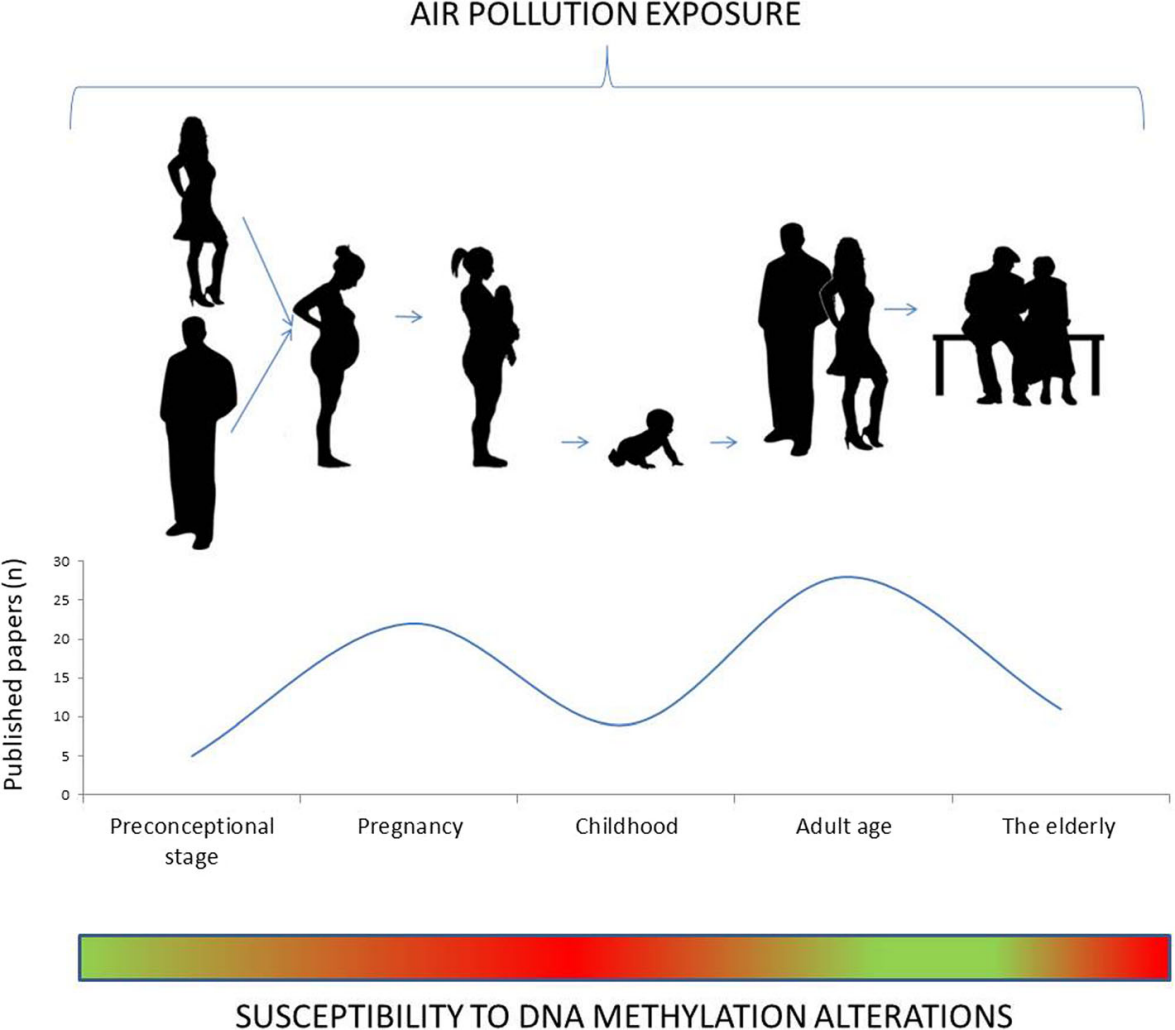
Effects of PM on DNA methylation throughout the lifespan. PM affects DNA methylation with an impact on health during all the life stages, from preconception to the elderly. The most studied life stages are pregnancy and the adult age. The reported evidences indicate that pregnancy, childhood, and the elderly can be considered hypersusceptibility windows (reported as red in the heat bar; green represents less impacted time windows)

As suggested in this review, many studies supported the hypothesis that PM could influence DNA methylation patterns. However, not all life stages are equally impacted: some life seasons such as preconception, intrauterine growth, early childhood, and older age are characterized by an increased susceptibility to the effects of PM (Fig. 2).

Taking together the literature here reviewed, it emerges that intrauterine life and childhood appear to be the life stages during which fewer studies have been conducted so far (Fig. 2). Nonetheless, they represent a very critical phase of life phases, during which epigenetic modifications may impact on development and growth of future adult individuals and may thus be associated with an increased risk of developing pathologies [41, 110, 111]. Therefore, there is the need to focus on epigenetic effects due to PM exposure during intrauterine life and childhood in future studies, in order to evaluate possible long-term effects on disease risks. Results may have a dramatic impact on prevention and public health policies.

## Data Availability

Not required

## Abbreviations

5-hmC: 5-Hydroxymethylcytosine
5-mdC: 5′-Methyl-deoxycytidine
BC: Black carbon
CAPs: Concentrated ambient particles
COPD: Chronic obstructive pulmonary disease
dC: Deoxycytidine
ETP: Endogenous thrombin potential
FEV1: Forced expiratory volume in 1 s
HEPA: High-efficiency particulate air
MS-MS: Tandem mass spectrometry
mtDNA: Mitochondrial DNA
NAS: Normative Aging Study
PAHs: Polycyclic aromatic hydrocarbons
PM: Particulate matter
UPLC: Ultra-pressure liquid chromatography
WHO: World Health Organization
